# Chronic Kidney Disease of unexplained cause (CKDx): a consensus statement by the Genes & Kidney Working Group of the ERA

**DOI:** 10.1093/ndt/gfaf092

**Published:** 2025-06-03

**Authors:** Jan Halbritter, Lucile Figueres, Albertien M Van Eerde, Giovambattista Capasso, Ewout J Hoorn, Tom Nijenhuis, Maria Vanessa Perez-Gomez, John A Sayer, Matias Simons, Stephen Walsh, Nikola Zagorec, Roman-Ulrich Müller, Emilie Cornec-Le Gall

**Affiliations:** Department of Nephrology and Medical Intensive Care, Charité Universitätsmedizin Berlin, Berlin, Germany; Nantes Université, CHU Nantes, INSERM, Center for Research in Transplantation and Translational Immunology, UMR 1064, ITUN, Nantes, France; Centre constitutif Filière OSCAR et Filière ORKID, CHU de Nantes, Nantes, France; Department of Genetics, University Medical Centre Utrecht, Utrecht, the Netherlands; Department of Medical Translational Sciences, University of Campania Luigi Vanvitelli, Naples, Italy; Biogem Scarl, Ariano Irpino, Italy; Department of Internal Medicine, Division of Nephrology and Transplantation, Erasmus Medical Center, University Medical Center Rotterdam, Rotterdam, the Netherlands; Department of Nephrology, Research Institute for Medical Innovations and Radboudumc Center of Expertise for Rare Kidney Diseases, Radboud University Medical Center, Nijmegen, the Netherlands; Department of Nephrology and Hypertension, Health Research Institute-Fundación Jiménez Díaz University Hospital, Universidad Autónoma de Madrid (IIS-FJD, UAM), 28040 Madrid, Spain; Department of Nephrology and Hypertension, RICORS2040, Hospital Universitario Fundación Jiménez Díaz, 28040 Madrid, Spain; Departamento de Medicina, RICORS2040, Facultad de Medicina, Universidad Autónoma de Madrid, Madrid, Spain; Biosciences Institute, Newcastle University, Central Parkway, Newcastle upon Tyne, UK; Renal Services, The Newcastle upon Tyne NHS Foundation Trust, Newcastle upon Tyne, UK; National Institute for Health Research Newcastle Biomedical Research Centre, Newcastle Upon Tyne, UK; Nephrogenetics Unit, Institute of Human Genetics, University Hospital Heidelberg, Heidelberg, Germany; London Tubular Centre, Department of Renal Medicine, University College London, London, UK; Service de Néphrologie, Hémodialyse et Transplantation Rénale, Centre de référence MARHEA, Filière ORKID, CHRU Brest, Brest, France; Faculty of Pharmacy and Biochemistry, University of Zagreb, Croatia & Department of Nephrology and Dialysis, Dubrava University Hospital, Zagreb, Croatia; Department II of Internal Medicine, Faculty of Medicine and University Hospital, University of Cologne, Cologne, Germany; Center for Rare Diseases Cologne, Faculty of Medicine and University Hospital Cologne, University of Cologne, Cologne, Germany; Cologne Excellence Cluster on Cellular Stress Responses in Aging-Associated Diseases (CECAD), Cologne, Germany; Service de Néphrologie, Hémodialyse et Transplantation Rénale, Centre de référence MARHEA, Filière ORKID, CHRU Brest, Brest, France; University Brest, Inserm, UMR 1078, GGB, Brest, France

**Keywords:** chronic kidney disease, CKD, CKD of unknown etiology, CKDx, genetic kidney disease

## Abstract

Chronic kidney disease of unexplained cause (CKDx) is a diagnosis of exclusion. With an estimated global prevalence of at least 16–20% among CKD patients, CKDx poses a significant challenge to the field. To date, there is no established consensus on the definition of CKDx. Additionally, guidance on the diagnosis and reporting of CKDx remains lacking. CKDx is characterized by the inability to identify a specific etiology after comprehensive diagnostic evaluation, including laboratory tests, imaging, and histological or genetic analyses. This condition encompasses diverse clinical scenarios, which vary depending on the availability of diagnostic resources across healthcare systems. Notably, as the diagnostic yield of genetic testing in CKDx ranges from 11 to over 30% in the literature, it has become an integral part of the diagnostic armamentarium for patients with CKDx. This consensus statement of the working group ‘Genes&Kidney’ of the European Renal Association proposes a definition of CKDx, along with recommendations for the diagnostic approach and diagnostic reporting standards, including guidance on genetic workup as a key tool in a large proportion of such cases. Improved reporting standards, including the systematic documentation of diagnostic tests performed, are essential

to avoid the negative therapeutic consequences of misdiagnoses, address the diagnostic gap in CKDx, and inform future research. By fostering a cause-directed approach, this work aims to enhance patient care and lay the foundation for further advancements in nephrology.

## DEFINITION OF CKDx

### ‘The greatest enemy of knowledge is not ignorance, it is the illusion of knowledge’, (Stephen Hawking)

Chronic kidney disease (CKD) is characterized by a gradual decline in kidney function over time due to a diverse set of underlying causes including acquired and genetic conditions. In many cases, however, a specific etiology cannot be identified, and clinicians often struggle to assign an appropriate diagnostic label. To address this gap, we propose the term CKDx (Chronic Kidney Disease of uneXplained cause) as a structured and standardized designation for cases in which no underlying cause can be determined after appropriate evaluation. International registries and cohort studies estimate the prevalence of CKDx to range between 16 and 20% of the total CKD population [1–3]. However, there is no consensus on a common definition of CKDx. Additionally, assignment of ‘diagnoses of convenience’, such as hypertensive nephropathy and vascular nephropathy, lead to systematic underestimation of true CKDx prevalence (Table 1) [4].

**Table 1:** Different commonly used terms that are non-specific diagnoses and benefit from further refinement (no claim to completeness).

Histological terms
● Focal segmental glomerulosclerosis (FSGS) ● Membranoproliferative glomerulonephritis (MPGN) ● Chronic interstitial nephritis (CIN) ● Thrombotic microangiopathy (TMA) ● Thin basement membrane nephropathy (TBMN) ● Nephrosclerosis or nephroangiosclerosis ● Tubulo-interstitial kidney disease ● Crystalline nephropathy
Clinical terms
● Familial (benign) haematuria ● Atrophic kidneys ● Hypoplastic kidneys ● Chronic glomerulonephritis ● Hypertensive nephropathy ● Vascular nephropathy ● Nephrocalcinosis ● Hyperuricemic nephropathy ● Congenital anomalies of the kidneys and urinary tracts (CAKUT)
Genetic terms
● Variant of uncertain (or unknown) significance (VUS) (ACMG class 3)

In 2012, the KDIGO guideline for CKD evaluation, classification, and management first introduced the CGA (**c**ause, **g**lomerular filtration rate, **a**lbuminuria)-classification framework [5, 6]. This framework served for risk-assessment of CKD mortality and cardiovascular morbidity by a three-dimensional categorical system including (i) underlying cause, (ii) estimated glomerular filtration rate (eGFR), and (iii) degree of albuminuria [7, 8]. Although the CGA-classification is widely adopted in clinical practice, the ‘cause’-component remains often neglected deserving more awareness among general practitioners and nephrologists. This critical point is further expanded upon in the 2024 Clinical Practice Guideline for the Evaluation and Management of CKD, emphasizing the evolving importance of the ‘cause’-component in CKD classification [9, 10]. At the same time, knowledge of CKD etiologies has increased tremendously. Advancements in sequencing technologies have greatly enhanced the diagnostic opportunities, enabling the identification of genetic components underlying various kidney diseases. Hundreds of new genes have been identified to be involved in CKD development, either in terms of risk alleles (e.g. *APOL1*) or as single-gene disorders involving the kidneys and/or the urinary tract [11].

**Figure 1:**
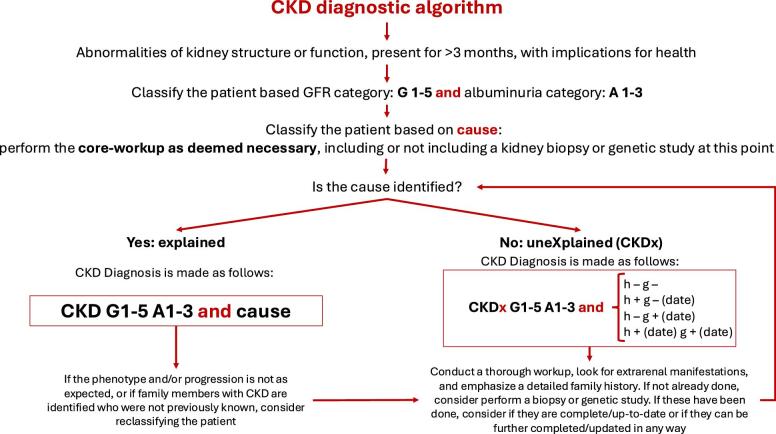
CKDx assessment flow chart (for guidance with genetic testing, see Supplementary data, Fig. S1).

CKDx is a subtype of CKD defined by the absence of an identifiable underlying condition (see Fig 1). By definition, CKDx is a diagnosis of exclusion following an updated and comprehensive diagnostic workup that includes core laboratory analytics, appropriate imaging studies, and specific diagnostic tests (e.g. serum antiPLA2R, ANCA, serum free light-chain, etc.). Depending on the clinical context, kidney histology and/or genetic testing should also be considered. The extent of diagnostic workup should reflect both the clinical presentation and the available diagnostic resources in a given healthcare setting. Full reporting of all diagnostic measures used prior to assigning a diagnosis of CKDx is crucial, both to ensure transparency and to enable future re-evaluation as diagnostic standards evolve (Supplementary data, Table S1). This definition of CKDx is designed to be applicable across healthcare systems, acknowledging that while an exhaustive workup is ideal, the threshold for assigning a diagnosis of CKDx must also consider limitations in diagnostic access.

Importantly, CKDx as suggested here, is distinct from a separate group of entities referred to by the acronym CKDu (CKD of unknown etiology), which describes endemic forms of CKD observed in specific geographic regions such as Central America, Sri Lanka, and India, primarily linked to occupational and environmental exposures, such as recurrent heat stress and dehydration [12]. These conditions are also known by various names including Mesoamerican nephropathy or chronic interstitial nephritis in agricultural communities (CINAC) [13].

Traditionally, kidney histology is considered the gold standard of CKD differential diagnosis. However, as CKD is frequently associated with co-morbidities such as hypertension or diabetes mellitus, these conditions are often assumed to be causal, and opportunities to perform a kidney biopsy may be missed for unjustified reasons. Evaluations in patients waiting for kidney transplantation yielded absence of kidney histology in about 40% of cases [14]. Furthermore, kidney biopsy, particularly when performed in CKD stage G4–5, is associated with an increased risk of haemorrhage and may yield unspecific results due to advanced fibrosis in the glomerular and tubulointerstitial compartment, or misinterpretation of histological patterns of injury as diagnoses, such as focal and segmental glomerulosclerosis (Table 1) [15, 16]. The emergence of non-invasive diagnostic tools, including immunological biomarkers (e.g. PLA2R or nephrin antibodies) or genetic testing, further questions the necessity of kidney biopsy in certain contexts [17]. In the light of these developments, nephrology should evolve from a model, in which the opportunity to a cause-directed personalized approach mainly relies on kidney histology (often limited to cases with proteinuria), to a model that enables access to the full potential of a rapidly growing diagnostic landscape including genetic approaches and state-of-the-art molecular histopathology. This will also help to apply an equivalent level of diagnostic rigor to CKD with bland urinary sediment, one of the major sources of cases with CKDx to date (Table 2). Importantly, defining CKDx as a basis to clarifying its cause is not only meaningful for selecting treatment strategies to maintain kidney function, but is still of clear importance after kidney failure has been reached. Actionability in the setting of kidney failure of unexplained cause includes many important aspects such as transplant decisions, evaluation of risk for recurrence on the kidney allograft, screening for extrarenal manifestations, family counselling and cascade screening when a monogenic cause is identified.

**Table 2:** Diagnosis pathways in exemplary real-world cases initially classified with CKDx and subsequently refined by genetic testing.

Case presentation	Diagnostic workup	Family history	Extrarenal features	Working diagnosis	Specific workup	Final diagnosis and retrograde phenotyping
M, 43y, AH, eGFR 34 mL/min/1.73 m^2^, P 1 g/day, E 0, CxG3bA3	Kidney USS: normal-sized kidneys, hyperechogenic parenchyma Kidney Bx: FSGS, thinner GBM (203±38 nm), podocyte foot processes preserved Tonal audiogram: normal	Father: KF (age 50y) Brother: KF (age 7y, kidney Tx) Son: vesicourethral reflux (age 1y)	Liver lesion induced by low-dose statin therapy	CKDx h+g–	Gene panel for FSGS: MYH9 c.1270C>T/p.Arg424Trp) (ACMG class 4)	MYH9-related disorder (MIM 155100) Reverse phenotyping: Mild occasional thrombocytopenia (143–153 × 10^6^/L) Elevated MPV (11.4 fL; normal <10.4) Ophthalmological: normal
M, 41y, AH, eGFR 50 mL/min/1.73 m^2^, P 0, E 0, CxG3aA1	Kidney USS: normal-sized kidneys (thinner cortex), loss of corticomedullary differentiation Kidney Bx: 40% of glomeruli globally sclerosed, chronic interstitial changes with mononuclear infiltration, GBM normal	Father: KF (age 51y) Paternal aunt: KF (age 58y)	Mild hyperuricaemia (407 µmol/L)	CKDx h+g– CKDx h+g+	Kidney panel and WES: negative MUC1-fs IHC: positive MUC1-VNTR: NM_001204286.1: c.428dupC (ACMG class 5)	ADTKD (autosomal dominant tubulointerstitial kidney disease) -*MUC1* (MIM 174000) Reverse phenotyping: No gout No other abnormalities found
M, 41y, AH, P 1 g/g, eGFR 108 mL/min/1.73 m^2^, recurrent fever episodes (∼1/month), CxG1aA3	Kidney USS: mildly enlarged kidneys with normal structure	Brother: KF due to renal amyloidosis (age 45y) Mother: died at age of 60y, unknown death cause	Bilateral deafness since childhood (cochlear implants)	CKDx h–g–	WES + virtual amyloidosis/autoinflammatory panel: NLRP3 c.1049 C>T/p.Thr350Met (ACMG class 5)	Muckle-Wells syndrome (MIM 191900) Appropriate treatment with IL-1 blockers led to disease remission
M, 59y, AH (age 49y), eGFR 33 mL/min/1.73 m^2^, P 1 g/day, DM type 2 (age 50y), CxG3bA3	Kidney USS: right kidney 14.1 cm with >10 cysts, left kidney 11 cm, >10 cysts—atypical polycystic kidney disease Liver USS: no liver cysts, cirrhosis?	Sister: polycystic kidney and liver disease (KF at age 52y, kidney Tx)	Liver disease/cirrhosis of unknow origin Gout	CKDx h–g– CKDx h–g+	Cystic gene panel: negative WES with virtual panel for kidney diseases: Biallelic TULP3 c.70C>T/p.Arg24* (ACMG class 5)	Hepato-reno-cardiac syndrome (MIM 619902) Reverse phenotyping: Liver biopsy performed at 49y of age: fibrosis F1
F, 49y, eGFR 20 mL/min/1.73 m^2^ (CKD known for 20y); AH, P 0.5 g/g, E 0, CxG4bA3	Kidney USS: Bilateral atrophic kidneys with several small-sized kidney cysts	Mother: eGFR 60 mL/min/1.73 m^2^ (age 74y) Brother: no kidney disease	Flat feet Learning difficulties (in school)	CKDx h–g–	WES with virtual panel for kidney: Monoallelic OFD1 c.1534C>T/p.Gln512* (ACMG class 5)	Oro-facio-digital syndrome 1 (MIM 311200) Reverse phenotyping: Micrognathia Delayed early development—cerebellar kinetic syndrome
F, 68y, second kidney Tx workup (CKD since age of 37y and KF at age of 49y), leukocyturia +, P 0, E 0, AH, CxG5Ax	Kidney USS: no kidney cysts Kidney Bx: chronic tubulointerstitial damage (EM not performed)—‘chronic tubulointerstitial nephropathy’	Mother: hypoacusia Sister: KF (81y), insulin dependent DM, neuropathy, epileptic myoclonus Two nieces (by sister): hypoacusia, kidney disease	Short stature (147 cm) Sudden onset of hypoacusia and insulin dependent DM after 1^st^ kidney Neurologic events: oral hypoesthesia, dysphagia	CKDx h+g–	Gene panel including mitochondrial genes: mt.3271 T>C in *MT-TL1* (level of heteroplasmy in blood 5%) (ACMG class 5)	MELAS (MIM 540000) Reverse phenotyping: Slightly elevated blood lactate Brain MRI: microvascular leukopathy; spectroscopy—high ventricular lactate level Neuropsychological testing: dysprosody and mild cognitive impairment

ACMG, American College of Medical Genetics and Genomics; AH, arterial hypertension; Bx, kidney biopsy; CK, creatin kinase; CKD, chronic kidney disease; CKDx, chronic kidney disease of unknown cause; DM, Diabetes mellitus; E, erythrocyturia; eGFR, estimated glomerular filtration rate; EM, electron microscopy; F, female; FSGS, focal segmental glomerulosclerosis; GBM, glomerular basement membrane; KF, kidney failure; M, male; MELAS, mitochondrial encephalopathy lactic acidosis; MPV, mean platelet volume; MRI, Magnetic resonance imaging; P, proteinuria; Tx, transplantation; USS, ultrasound scan; WES, whole exome sequencing.

Box 1. **Statements and practice points on the definition of CKDx**

**Statement 1.1:**

The ‘cause’-component of the KDIGO CGA-classification of CKD deserves special attention considering its impact on patient counselling, prognosis, cardiovascular risk stratification and therapeutic strategies.

**Practice point 1.1:**

CKDx is a diagnosis of exclusion. We suggest classifying the cause of CKD as CKDx in all cases in which a specific cause cannot be defined in a given healthcare system after reasonable diagnostic workup; taking into account potential limitations in access to and availability of modern diagnostic technologies.

**Statement 1.2:**

Naming causes with low diagnostic certainty or unspecific terms, including histological injury patterns, may lead to unnecessary, potentially harmful or missed therapies and is likely to discourage further diagnostic workup, even as additional diagnostic approaches become available. If the diagnostic processes do not identify a clear cause, the condition should be classified as CKDx.

**Statement 1.3:**

Nephrology should evolve to a cause-directed approach in all cases of CKD.

## WORKUP OF CKDx

If a patient's kidney disease is classified as presumed CKDx, we assume that a comprehensive general clinical workup has been conducted to identify all common causes of CKD (Supplementary data, Table S1). If, at this point, the diagnosis is not clarified, the diagnostic workup is usually completed by specialized non-invasive diagnostic tools targeted to previous findings or patient characteristics (Supplementary data, Table S1). In principle, both kidney biopsy and genetic exams can be warranted at any step during the procedure. However, both approaches—while not necessarily always essential—must be considered to extend the diagnostic efforts in an obligatory manner as long as a diagnosis has not been achieved with a high degree of certainty. Once CKDx is established, the objective is to identify rare types of kidney diseases, including inherited conditions, that may underlie unspecific CKD and were not evident during the initial diagnostic workup. The comprehensiveness of these workups mainly depends on the patient's medical history, availability of diagnostic tools, and the clinician's experience. However, not all suggested tests should be routinely performed, as they require appropriate indication and interpretation and may be costly and time-consuming.

**Figure 2:**
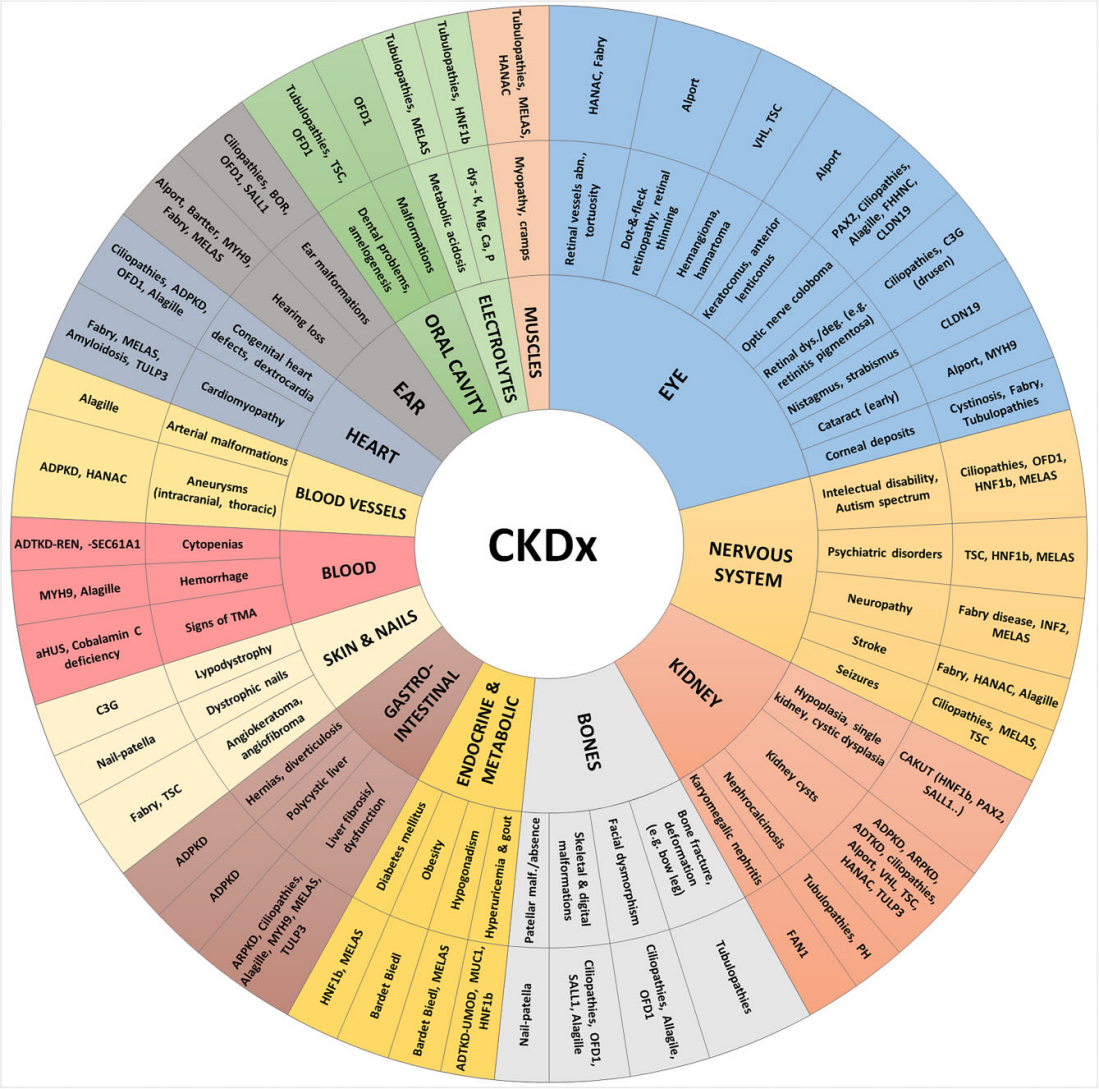
Sunburst-like plot showing possible extrarenal and renal features associated with genetic kidney conditions. (Of note: pie-size does not reflect real frequency; not all possible extrarenal features are listed here nor all genetic kidney diseases are covered.) Abbreviations: ADPKD, autosomal dominant polycystic kidney disease; ARPKD, autosomal recessive polycystic kidney disease; ADTKD, autosomal dominant tubulointerstitial kidney disease; BOR, branchio-oto-renal; CAKUT, congenital anomalies of the kidneys and urinary tract; C3G, C3 glomerulopathy; HANAC, hereditary angiopathy with nephropathy, aneurysms and cramps (*COL4A1* gene); *HNF1b*, gene coding for hepatic nuclear factor 1-beta; *FAN1*, FANCD2/FANCI-associated nuclease 1; MELAS, mitochondrial encephalopathy, lactic acidosis, stroke-like episodes; *MUC1*, gene coding for mucin 1; *MYH9*, gene coding for myosin heavy chain 9; OFD1, orofaciodigital syndrome 1; PH, primary hyperoxaluria; *REN*, gene coding for renin; *SALL1*, gene coding for spalt-like transcription factor 1 (Townes-Brockes syndrome); *SEC61A1*, gene coding for Sec61 subunit alpha isoform 1; TMA, thrombotic microangiopathy; TSC, tuberous sclerosis complex; *TULP3*, gene coding for TUB like protein 3 (hepatorenocardiac syndrome), *UMOD*—gene coding for uromodulin; *VHL*, von Hippel Lindau disease, *WT1*, Wilms’ tumour suppressor gene. Explanations: 1. The term *‘ciliopathies’* refers to broad spectrum of multisystemic disorders originating from primary cilia disorder with kidney, nervous system, skeleton, eye and other organs affection. (e.g. nephronophtysis, Senior-Loken syndrome, Joubert syndrome, Alström syndrome, Bardet-Biedl, etc.). Ciliopathies may include disorders like ADPKD, ARPKD and OFD1-related disease, although in this figure they are shown separately. 2. The term *‘tubulopathies’* refers to broad spectrum of diseases caused by renal tubular disfunction resulting in electrolyte (K, P, Ca, Mg, Na, Cl) and/or renal acid-base disorders with common systemic implications (e.g. Gitelman syndrome, Bartter syndrome, renal hypophosphatemia, familial hypomagnesaemia with hypercalciuria and nephrocalcinosis, etc.).

We suggest revisiting the evaluation process with a dedicated diagnostic re-evaluation consultation. Such an approach entails a reassessment of all diagnostic elements directly from their original source rather than relying on summaries. This process begins with a thorough reassessment of the patient's history and physical examination, focusing specifically on aspects indicative of rare kidney diseases, including extrarenal features (Fig. 2 and Supplementary data, Table S1). In this context, it is important to consider that while individual rare kidney diseases are infrequent by nature, together they make up a considerable fraction of CKD cases. A detailed and chronological timeline of clinical events should be reconstructed, integrating findings from the patient's history, physical examination, and prior investigation. Such a comprehensive re-evaluation can reveal overlooked or previously misinterpreted findings, even leading directly to a definitive diagnosis in some cases and should be proposed to every patient with CKD.

A positive family history, age at onset, disease course and the presence of extrarenal features (see Fig. 2) can suggest an inherited kidney disease. However, none of these factors are required for diagnosing a genetic kidney disease, and importantly, a negative family history does not exclude genetic CKD.

Family history may directly be assessed with the help of a genetic pedigree (see Supplementary data, Table S2 for available tools). It is beneficial for both the patient and the clinician (nephrologist or clinical geneticist) to thoroughly include all first- and second-degree relatives and to ask for possible consanguinity and any instances of premature death. Instead of merely inquiring whether the patient has relatives who have undergone kidney transplantation or dialysis, it is crucial to specify the type of kidney disease whenever possible as well as the age at onset of kidney replacement therapy, extrarenal manifestations associated with genetic kidney disease and age at onset of extrarenal disorders (Supplementary data, Table S1), thus increasing the likelihood of not missing clues for hereditary disease. Informing patients beforehand about the type of information required may help ensure completeness and prepare patients to questions that may evoke emotional responses (e.g. fetal death or sudden death in a relative).

Characteristics of the patient's birth may be relevant, particularly in detecting prematurity or abnormal growth or development, which can affect nephron number and development [18–20], or neonatal acute kidney injury. Scholastic achievements and current or former employment or vocations of the patient can indirectly indicate potential intellectual disabilities, impaired mental health, or disabling symptoms. A patient's professional background may also suggest prior medical screenings (e.g. military or insurance), which can provide valuable information such as kidney function tests or urinalysis, aiding in the assessment of kidney disease duration.

While a kidney ultrasound scan is typically already included in the general workup, additional imaging may be required depending on the nature of the disease, since ultrasound is not always sensitive enough to detect small size cysts and accurately quantify nephrocalcinosis. Magnetic resonance imaging (MRI) shows the best sensitivity to detect small cysts. For kidney stones and/or nephrocalcinosis, non-contrast enhanced computed tomography is preferred in adults [21].

After gathering and interpreting all diagnostic information tailored to the patient's individual characteristics, consideration should be given to performing a kidney biopsy, genetic testing, or both (Fig. 1). If these procedures were previously conducted in the general workup, it is essential to assess whether they were comprehensive and meet current standards, especially if a significant amount of time has passed since for example genetic testing, to ensure the patient receives the most relevant and thorough evaluation (e.g. electron microscopy if not performed initially, specific immunohistochemistry, or extension of genetic testing towards an exome/genome or a mitochondrial disease panel, see Genetic Testing). Effective collaboration between nephrologists, nephropathologists and geneticists is crucial for CKDx investigation, particularly given the recent advancements in genetic testing modalities, availability and sensitivity (see Supplementary data, Fig. S1).

Box 2. **Statements and practice points on the workup of CKDx**

**Statement 2.1:**

A systematic diagnostic approach to CKD and standardized reporting of findings helps to secure full use of all diagnostic opportunities required to reach the diagnosis of exclusion CKDx.

**Practice point 2.1:**

A structured family history should be obtained for all patients with CKD and specific extension of the information (e.g. towards extrarenal manifestations) should be considered depending on individual case characteristics.

## REPORTING OF CKDx

If the diagnosis re-evaluation process does not result in the identification of a clear cause, the condition should be reported as CKDx. Providing detailed information on prior diagnostics is crucial to enable others to assess diagnostic certainty and select appropriate additional tools based on availability. To meet this necessity and to underline the importance of kidney histology and genetic testing in the differential diagnosis of CKD, we propose a novel reporting system for CKDx. This new classification provides structured information on the availability of genetic and histological results including the year when the test was performed. In this context, ‘h’ represents histology and ‘g’ genetic testing, with a plus sign (+) indicating that a test has been performed (with the date of performance added), while a minus sign (-) indicates that the test has not been performed. For example, CKDx G4 A3 (h–g+(2024)) describes a patient in whom a kidney biopsy was not performed, but genetic workup was conducted in 2024. The genetic exam did not provide a diagnosis, which is why the patient's CKD is classified as CKDx. Additionally, clinical, histological, or genetic findings which—while still insufficient for a definitive diagnosis—are nonetheless important to assess the case and completeness of diagnostic data, should be included (Table 1) and important technical characteristics of the genetic exam (i.e. the use of panel, exome-based panel, exome or genome) and biopsy results should be specified if available. The classification workflow is presented in Fig. 1. Several clinical vignettes illustrating the diagnostic workflow are included in Table 2.

The diagnosis CKDx avoids wrong treatment strategies based on low-certainty diagnoses of convenience and emphasizes the special responsibility of the nephrologist to update the workup or seek expert advice to do so, according to constant evolutions in diagnostic standards during the follow-up of the patient.

Box 3. **Statements and practice points on the reporting of CKDx**

**Statement 3.1:**

In cases of CKDx, we suggest extending usual reporting of the diagnosis (based on the KDIGO CGA-classification) by providing structured information on the availability of genetic and histological results including the year when the test was performed. In this regard, ‘h’ represents histology and ‘g’ genetic testing, with a ± sign indicating whether the respective test was performed and the performing date mentioned (Fig. 1).

**Practice point 3.1:**

In addition to the h/g-extension of CKDx reporting, information crucial to interpreting the workup should be provided. It includes the genetic testing modality used (e.g. gene panel, WES), year when it was performed and biopsy evaluation (e.g. electron microscopy) (Fig. 1).

**Practice point 3.2:**

Although this consensus statement specifically provides guidance for CKDx, reporting the availability of genetic and biopsy results may also be considered for other CKD cases.

## GENETIC TESTING IN KIDNEY DISEASE: CLINICAL CONCEPTS

While kidney histopathology remains a central tool to diagnostic algorithms in CKD and will gain further importance considering upcoming molecular approaches based on kidney tissue, the greatest changes in diagnostic algorithms over the last decades have been implemented in the area of genetic testing. This includes the increasing awareness of the large contribution of genetic causes to CKD as well as upcoming and guideline-supported genetics first approaches depending on patient characteristics. This comes with an important need for guidance regarding strategies to genetic testing. The following paragraph explores this area in respect of CKDx.

### Indications for genetic testing

The advent of massive parallel sequencing (MPS), also referred to as Next Generation Sequencing (NGS), has greatly expanded diagnostic capabilities and led to the discovery of numerous novel genes associated with kidney disease. Many genes or even entire genomes can now be analysed at once using gene panels or whole-exome sequencing (WES)/whole-genome sequencing (WGS). In the context of CKDx at a relatively young age (e.g. before 60 years), genetic testing is increasingly regarded as a standard component of the diagnostic workup. Consequently, decisions not to pursue genetic testing in such cases now require explicit justification. There are patient and family characteristics hinting at a genetic diagnosis (discussed above), but strictly adhering to them will lead to missing diagnoses that could have major impact on patients and families [11, 22]. Almost all studies on state-of-the-art genetic testing in large CKD-cohorts have revealed diagnostic yields of at least 10% regardless of the clinical presentation or underlying suspicion of genetic disease [23]. However, diagnostic yield of genetic testing varies widely across studies, largely depending on inclusion criteria—such as younger age at onset, presence of syndromic features, or positive family history—which enrich cohorts for a suspected genetic etiology (Supplementary data, Table S3). Additional reasons to perform genetic testing include: (i) whether a molecular diagnosis would influence a patient's reproductive decisions, such as opting for preimplantation or prenatal genetic testing; (ii) if a patient has relatives that would benefit from knowing about a monogenic disease (i.e. in context of closer monitoring, organ donation or reproductive decisions); (iii) when invasive diagnostic procedures can be prevented; (iv) when specific therapies would be indicated, or discontinued (e.g. immunosuppressive therapies in FSGS); (v) when a definitive diagnosis would help to relieve patient anxiety.

### How to genetically test patients with CKDx

It is important to emphasize that there are many local variations in the organization of nephrogenetic care. Whenever feasible, priority should be placed on establishing or connecting with multidisciplinary infrastructures, centers of expertise, or colleagues who function as consultable hubs [24, 25] (Supplementary data, Table S2). While access to genetic testing varies between healthcare systems, growing evidence suggests that early implementation of genomic testing can be cost-effective or even cost-saving. A recent real-world study by Becherucci *et al*. demonstrated a 41% actual cost reduction using a multistep diagnostic workflow incorporating early exome sequencing [26]. Similarly, two studies reported favorable cost-effectiveness outcomes, particularly for glomerular diseases and in pediatric populations [27, 28]. These studies only focussed on cost-effectiveness in index cases, without accounting for the potential benefits in their family members. These findings support the strategic and timely integration of genetic testing into CKDx workup pathways. Cost-effectiveness is expected to increase with technological advances allowing to consider genetic testing at earlier steps in these pathways in the future.

There are various genetic tests available, and it is essential to understand the distinctions between the sequencing method used to generate data and the specific analyses performed. Terms like gene panel, exome, and genome are sometimes employed interchangeably which can create confusion. For instance, a kidney disease gene panel can be a virtual panel applied on sequencing data generated from exome sequencing or genome sequencing (i.e. only a selected portion of WES/WGS data is used); or obtained after targeted capture of a selection of genes (i.e. custom MPS panel). A ‘clinical exome’ examines the coding portion of the genes associated with any known human disease (<30% of the genes). Using a large kidney disease gene panel as an initial testing approach for an index patient with CKDx is a valuable strategy, provided they have not had prior broad testing. Resources such as PanelApp provide curated gene lists for specific disorders, and the current kidney disease gene panel includes more than 600 genes (Supplementary data, Table S2) [29–32]. The scope of analysis has significant implications for patient care. The broader the analysis, the higher the chance of incidental findings, which may impact the care of the patient. There is a key role for the nephrologist in holistic and detailed phenotyping as this information is used in variant classification. This process can be streamlined through digital phenotyping tools that help translate clinical signs and symptoms into machine-readable codes, such as the Human Phenome Ontology (HPO) [33]. While this step is sometimes overlooked by clinicians, it can significantly enhance the diagnostic yield of genetic testing by optimizing bioinformatic variant prioritization pipelines (i.e. highlight variants in genes associated with the HPO terms supplied by the prescriber).

### Post-test considerations: Understanding variant classification

Careful consideration of the patient's phenotype and disease severity is essential when interpreting genetic variants. The American College of Medical Genetics and Genomics (ACMG) criteria currently guide the classification of variants into five classes using a specific scoring system: classes 4 and 5 are likely pathogenic and pathogenic (LP/P), respectively, while classes 1 and 2 are benign and likely benign [34]. Accurate classification and potential reclassification are important, as classifications can change over time. Once a variant is classified as pathogenic (class 5), it usually remains so; however, this does not necessarily imply causality in an individual patient, particularly when the inheritance pattern does not fit or in cases of reduced penetrance (e.g. *SLC34A1* variant). Therefore, multidisciplinary evaluation and reverse phenotyping are essential to accurately interpret such variants.

Variants of uncertain significance (VUS; class 3) are ideally only reported when there is a high likelihood that they might be causal for the disease and realistic chance to reclassify them as diagnostic (LP/P) through further information resulting from for instance reverse phenotyping, segregation analyses or functional assays [34, 35]. VUS should not be considered diagnostic. These variants should be periodically reviewed, perhaps every two to three years, as updates in annotation may lead to reclassification as pathogenic or benign. Re-evaluation is a shared responsibility between clinicians and diagnostic laboratories, as both evolving clinical data and new evidence from genomic databases or functional studies may contribute to variant reclassification over time. In cases of VUS, discussing findings in a multidisciplinary meeting or expert setting is recommended to ensure collective interpretation and decision-making until reclassification is performed. Caution in interpretation is equally important for pathogenic or likely pathogenic variants as pathogenicity does not necessarily imply causality in a given patient. In fragmented health systems, where prior diagnostic data may be incomplete or inaccessible, there is a risk that genetic findings—if not supported by clinical features—may be misinterpreted. Reverse phenotyping is therefore essential, ensuring that genetic results are interpreted in the context of a carefully assessed clinical presentation rather than in isolation.

In the clinical setting, polygenic risk scores are not yet used in nephrology. However, some risk alleles, such as *APOL1* risk alleles, are important for understanding the genetic basis of the disease and may have therapeutic implications. *APOL1* should be routinely included in broad genetic panels used for CKDx, as clinician-assigned ancestry may be imprecise or not explicitly documented, and genetic admixture may complicate assessment. Inclusion is particularly important for patients from populations where *APOL1* risk alleles are enriched (e.g. West-African ancestry), especially when presenting with clinical features such as proteinuria or focal segmental glomerulosclerosis (FSGS) on biopsy [36].

### Post-test considerations: negative or inconclusive results

While our understanding of genetic variants and detection technologies grows, it remains limited; therefore, a negative test does not exclude a genetic cause. Interpretation of negative test results must consider the specific gene selection used, the limitations of the technology, and the year the test was performed. The genetic analysis report must clearly state the analyses performed, including whether copy number variation (CNV) was conducted, which allows the detection of deletions, duplications and large rearrangements. The evidence for robust gene-disease relationships and variant-disease associations is regularly curated by experts in the field [29]. In cases with negative results, particularly when hereditary disease is suspected, periodic review and consideration of test expansion to fill diagnostic gaps in a multidisciplinary setting is essential. Common diagnostic gaps should be systematically addressed: for example, in cases of CKDx with an ADTKD-like presentation, it is essential to specify if the *MUC1* VNTR region was analysed, potentially using VNtyper [37]. Furthermore, in individuals with atypical HUS, particular attention should be paid to the methods used to investigate hybrid gene formation within the CFHR gene cluster. While multiplex ligation-dependent probe amplification (MLPA) is commonly used—typically in addition to exome sequencing or large gene panels—nanopore sequencing technology, although not yet widely implemented in routine diagnostics, offers a promising ‘all-in-one’ approach. It enables the simultaneous detection of sequence variants and structural rearrangements within a short timeframe, potentially providing actionable information to guide therapeutic decisions, even in critical care settings [38]. In another context, if the clinical presentation or inheritance pattern suggests a mitochondrial disease, the report should indicate whether mtDNA was captured. Such examples are highlighted in Table 2. Currently, advanced methods such as long-read sequencing and RNA sequencing are reserved for specific cases. For further details, please refer to Supplementary data, Fig. S1. With advances in techniques, the standard of nephrogenetic care is continuously evolving. This also comes with the need of updating guidance documents since advice such as the content of this paragraph may be outdated more rapidly than previously considered.

Effective genetic testing in nephrology requires a comprehensive understanding of the clinical scenarios that warrant testing, appropriate methods, result interpretation within the context of available genetic panels and patient-specific factors, and regular re-evaluation based on advancements in genetic techniques and knowledge. Striking a balance between simplicity and accuracy is essential: overly simplifying guidelines for general nephrologists risks misinterpretation and incomplete testing. However, general nephrologists play a key role in recognizing patients who may need genetic testing, characterizing their phenotype, and initiating first-tier tests, ensuring barriers to genetic testing are as low as possible. Again, this underlines the need for (interdisciplinary) discussion of CKDx cases with centers of expertise.

Box 4. **Statements and practice points on genetic testing in CKDx**

**Statement 4.1:**

Genetic testing should be considered in all cases of CKDx. The expected diagnostic rate of genetic testing depends on the presence of several factors in the patient history. In individuals under 60 years of age, it is increasingly regarded as a standard diagnostic approach; omission of testing in this population should be supported by a clear clinical rationale.

**Statement 4.2:**

The genetic testing modality and genetic care infrastructure may vary between healthcare systems. The minimum required selection of genes to test in CKDx is a comprehensive kidney disease gene panel consisting of genes with moderate to definitive evidence for gene–disease association, as curated in standardized resources (e.g. ClinGen, PanelApp, see Supplementary data, Table S2).

**Statement 4.3:**

An inconclusive or negative genetic test does not exclude a genetic cause.

**Statement 4.4:**

Identification of a pathogenic or likely pathogenic variant does not necessarily indicate that the variant is causal for the patient's kidney disease. Interpretation must take into account the clinical phenotype and inheritance pattern.

**Practice point 4.1:**

VUS should not be considered as conclusive diagnostic findings and require further discussions. Contact with expert centres is key. Reverse phenotyping, cosegregation analysis, and/or functional assays can help to reclassify such variants as likely pathogenic (or likely benign)

**Practice point 4.2:**

If genetic testing does not identify a causal variant, patients should be informed that a genetic cause has not been definitively excluded. Further interpretation may include referral to an expert centre, expansion of testing, or re-evaluation of previous findings. Periodic re-assessment is recommended as new clinical or genetic information may emerge over time.

## CONCLUSION

CKDx represents a significant challenge in nephrology and is an important area for nephrologists to both get a better understanding of and to move the field towards cause-directed personalized approaches. Advancing our understanding of CKDx requires a thorough examination of clinical, histopathological and genetic investigations, harbouring key opportunities for improved patient care and research through clear disease definition. Moving forward, defining CKDx will rely on improved diagnostic techniques, including genetic testing, registries for patients with CKDx and a deeper understanding of potential causes. We hope this consensus statement will help the field transition from a pre-paradigmatic era, where FSGS and hypertensive nephropathy are considered primary diagnoses, to a framework where they are labelled as CKDx and in which information on the diagnostic data available is provided in a structured fashion (e.g. h+g– or h–g–). This transition would also be the basis to periodic revisiting of diagnoses. Recognizing the absence of a definitive diagnosis is a critical first step toward establishing precision diagnostics.

The successful implementation of this classification will require adoption by stakeholders, scientific societies, and academic bodies, integrating this terminology into the classification of nephropathies. Eventually, a revision of current international diagnostic codes might be necessary to reflect this evolving paradigm.

## Supplementary Material

gfaf092_Supplemental_File

## Data Availability

No new data were generated or analysed in support of this research.
