# Supplementary material for: Chronic Kidney Disease of unexplained cause (CKDx): a consensus statement by the Genes & Kidney Working Group of the ERA
Source: Nephrol Dial Transplant. 2025 Jun 3;40(12):2390–400. doi: 10.1093/ndt/gfaf092 (PMC12709129; doi:10.1093/ndt/gfaf092)
Supplement: gfaf092_Supplemental_File [file gfaf092_Supplemental_File.docx]

**A structured framework for Chronic Kidney Disease of unexplained cause (CKDx) and guidance for genetic testing: a consensus statement by the Genes&Kidney Working Group of the European Renal Association**

**Supplementary data**

**Table S1:** General and targeted clinical workup in CKDx

|  | General workup | Targeted workup |
| --- | --- | --- |
| Personal  Medical History | - Age of presentation - Kidney stones - Ask for extrarenal symptoms: neurological (seizures, neuropathy, strokes, developmental delay), eye, deafness, heart, bone, hypertension, diabetes, exercise intolerance, fertility | - Birth (premature, birth weight, admission NICU) - Miscarriage - Type of education and current profession |
| Pedigree | - Draw the pedigree: family history (3 generations, consanguinity, family members with kidney failure, other hereditary diseases, causes of death) | - Review the initial pedigree and enrich it with new information - Suggest screening relatives (UPCR, eGFR, ultrasound) whenever possible |
| Clinic | - BMI - RR - Height | Look for extrarenal manifestations:   - Syndromic features - Height - Eye - Ear - Teeth - Skin |
| Laboratory | - Historic course of eGFR, urine sediment and albuminuria/proteinuria | - Blood: acid-base, lactate, uric acid, magnesium, liver enzymes, CPK - Urine: glucose (without hyperglycaemia and SGLT2i), LMWP (A1MG), urine microscopy if available - Immunology work-up: ANCA, ANA, Complement Protein electrophoresis and κ and λ light chains |
| Imaging | - Ultrasound | - Consider MRI in selected cases - CT for kidney stones, nephrocalcinosis |
| Histology |  | - First step: Kidney biopsy, general histology - Consider electron microscopy in specific settings - Specific staining (e.g. DNAJB9 in fibrillary GN) - Re-examining the initial kidney biopsie(s) (sometimes performed several years before) is important whenever possible |
| Genetics |  | Depending on the setting:   - Gene-panel testing - Whole-exome sequencing - Whole-genome sequencing |

Abbreviations: ANA, antinuclear antibody; ANCA, antineutrophil cytoplasmic antibodies; BMI, body mass index; CPK, creatine phosphokinase; CT, computer tomography; eGFR, estimated glomerular filtration rate; GN, glomerulonephritis; LMWP, low molecular weight protamine; MRI, magnetic resonance imaging; NICU, neonatal intensive care unit; SGLT2i, sodium glucose linked transporter 2 inhibitor; UPCR, urine protein-creatinine ratio.

**Table S2**: Helpful resources and tools with regard to CKDx

| General clinical genetics/ Pre- and post-test counselling / Guidance on report reading | |
| --- | --- |
| NHS genomics education programme* | genomicseducation.hee.nhs.uk/education/?swoof=1&product_cat=videos |
| NHGRI resources* | genome.gov/About-Genomics/Educational-Resources |
| Melbourne Genomics guide to genomics* | kidneygenomics.org.au (targeted towards (paediatric) nephrology genetics) |
| Pedigree drawing | |
| Pedigree drawing tutorial | genomicseducation.hee.nhs.uk/taking-and-drawing-a-family-history/ |
| Drawped | genecascade.org/ped-cgi/pedigree.cgi |
| Quickped | https://magnusdv.shinyapps.io/quickped/ |
| Progeny** | progenygenetics.com/online-pedigree/ |
| CeGAT pedigree software** | https://cegat.com/de/diagnostik/allgemeine-informationen/stammbaumerstellung/ |
| Invitae** | https://familyhistory.invitae.com/login/?next=/ (account necessary) |
| Check if pedigree building tools are provided for in your electronic health record | |
| Symptoms annotation | |
| SAMS | https://www.genecascade.org/sams-cgi/index.cgi |
| Disease and/or gene information | |
| Orphanet | orpha.net/en/disease |
| ERKNet | erknet.org/disease-information and youtube.com/@erkucation/videos |
| GeneReviews | ncbi.nlm.nih.gov/books/NBK1116/ |
| Omim | omim.org/ |
| GeneCards | genecards.org |
| Aids for assessing gene panel content | |
| PanelApp UK | panelapp.genomicsengland.co.uk/ |
| PanelApp Australia | https://panelapp.agha.umccr.org/ |
| Clingen | www.clinicalgenome.org/ |
| You can compare with the panel content of academically or commercially available kidney gene panels | |

* be aware of local, ethical, legal, organizational differences

** offered by a company with commercial interests

**Table S3**: Overview of studies examining the diagnostics yield of genetic testing in kidney disease

| Reference | Population Concerned | N (CKDx/ Total Number) | Technique Used | Diagnostic Yield |
| --- | --- | --- | --- | --- |
| Schrezenmeier et al., Genetics in Medicine 2021 [1] | Single-center (Germany). Patients on kidney transplant waiting list with CKDx and under 40 years at discovery of CKD | 115 | Targeted sequencing of 600 genes | 20.6%, including 11.1% confirmed diagnoses |
| Ottlewski et al.,  Kidney Int 2019 [2] | Single-center (Germany). Patients on kidney transplant waiting list with CKDx | 57 | OMIM panel sequencing (4813 genes), targeted analysis of 209 genes | 12% |
| Connaughton et al., Kidney Int 2019 [3] | Multicenter (Ireland). CKDx with family history of CKD or extra-renal features | 34 | Targeted sequencing of 478 genes | 47% |
| Lata et al.,  Annals of Internal Medicine 2018 [4] | CKDx with positive family history (56%) or age at onset/extra-renal signs suggestive of genetic origin | 83/92 | Targeted sequencing of 287 genes | 17% |
| Groopman et al.,  NEJM 2019 [5] | AURORA clinical trial (haemodialysis) and Columbia CKD cohort | 281/3315 | Exome sequencing followed by targeted analysis of 625 genes | 17.1% |
| Lazaro-Guevara et al., Am J Nephrol 2021 [6] | Single-center (Utah Kidney Study). Patients followed in nephrology for CKD or on dialysis | 206 (97 NND and 109 ND) | Targeted sequencing of 345 genes | NND: 19% confirmed diagnoses; ND: 22% |
| Mallawaarachchi et al., CJASN 2024 [7] | Multicenter (Australia). CKDxG5, age under 50. Excluded probable diabetic or renovascular nephropathies, and cystic/glomerular family histories | 100 | Genome sequencing followed by targeted analysis of a 388-gene nephrome. Mendeliome and mitochondrial variants analysed if nephrome was negative. | 25% |
| Blasco et al.,  AJKD 2024 [8] | Multicenter (Spain). CKDx G5, age under 45 | 818 | Targeted sequencing of 529 genes | 24.8% |
| Robert et al.,  CKJ 2024 [9] | Multicenter (France). CKDx G1-G5 | 230 | Exome sequencing followed by targeted analysis of 675 genes | 32.6% |
| De Haan et al.,  NDT 2024 [10] | Multicenter (Netherlands). CKDx G3 and higher (eGFR <60mL/min) with onset <50 years | 340 | Exome sequencing followed by targeted analysis of either 256 or 495 genes | 17% |
| Leenen et al.,  NDT 2022 [11] | Single center (Germany). Waitlisted patients fulfilling one of the following criteria: (i) unclear aetiology (ii) clinically suspected genetic kidney disease (iii) positive family history | 58 | Targeted sequencing of 479 nephropathy-associated genes | 33% |
| Becherucci et al.,  JASN 2023 [12] | Multicenter (Italy). Selection criteria: resistance to treatment OR family history OR extrarenal involvement OR CAKUT and CKD stage≥ 2 OR at least two cysts in each kidney OR hyperechoic kidneys/nephrocalcinosis OR persistent metabolic abnormalities | 476 | Exome sequencing and targeted analysis of kidney disease-associated genes | 67% (including 48% confirmed diagnosis) |


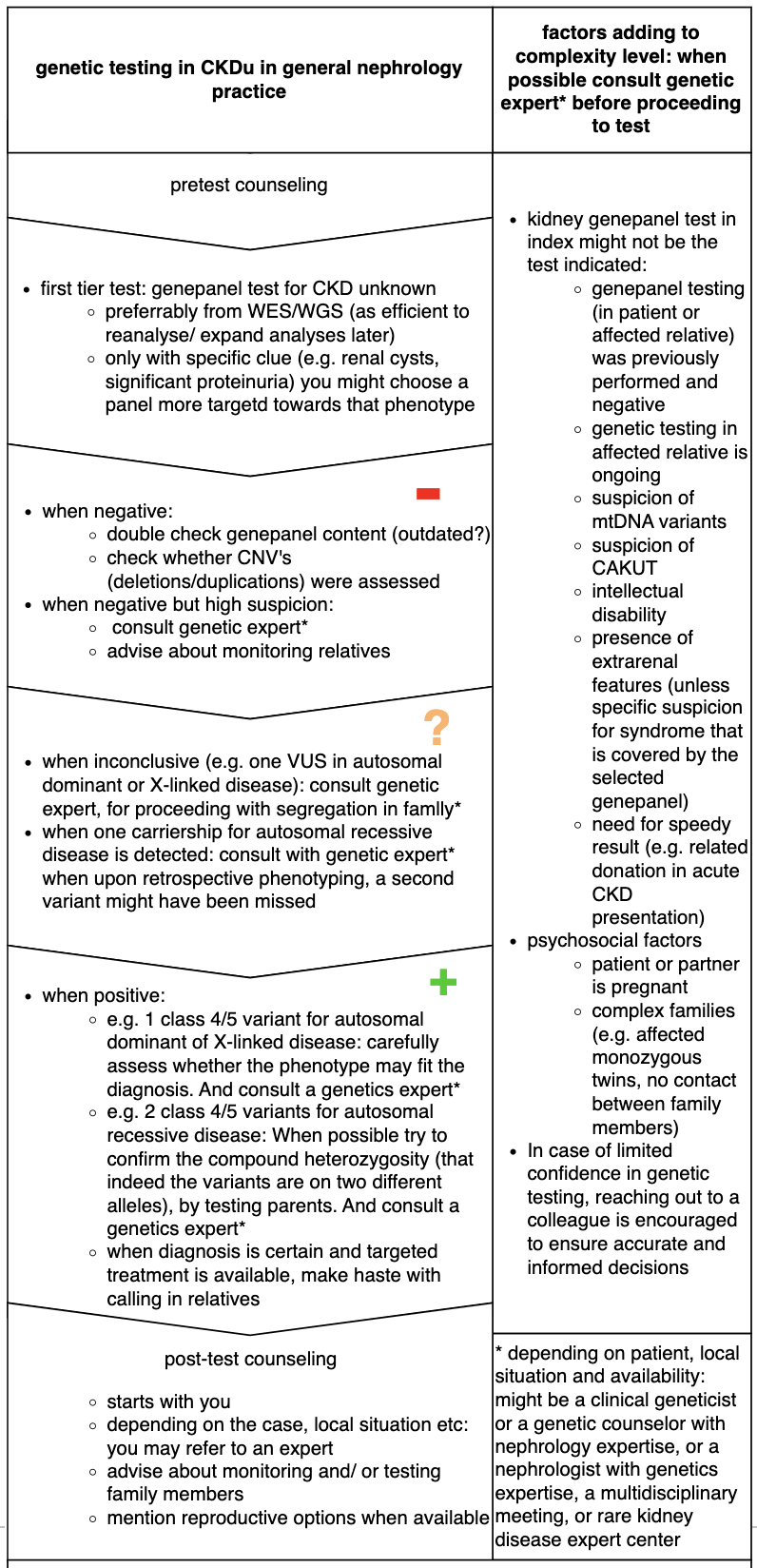


**Figure S1:** Flowchart for genetic testing in CKDx

**References**

1. Schrezenmeier E, Kremerskothen E, Halleck F et al. The underestimated burden of monogenic kidney disease in adults waitlisted for kidney transplantation. *Genet Med* 2021;**23**:1219-24.

2. Ottlewski I, Münch J, Wagner T et al. Value of renal gene panel diagnostics in adults waiting for kidney transplantation due to undetermined end-stage renal disease. *Kidney Int* 2019;**96**:222-30.

3. Connaughton DM, Kennedy C, Shril S et al. Monogenic causes of chronic kidney disease in adults. *Kidney Int* 2019;**95**:914-28.

4. Lata S, Marasa M, Li Y et al. Whole-Exome Sequencing in Adults With Chronic Kidney Disease: A Pilot Study. *Ann Intern Med* 2018;**168**:100-9.

5. Groopman EE, Marasa M, Cameron-Christie S et al. Diagnostic Utility of Exome Sequencing for Kidney Disease. *N Engl J Med* 2019;**380**:142-51.

6. Lazaro-Guevara J, Fierro-Morales J, Wright AH et al. Targeted Next-Generation Sequencing Identifies Pathogenic Variants in Diabetic Kidney Disease. *Am J Nephrol* 2021;**52**:239-49.

7. Mallawaarachchi AC, Fowles L, Wardrop L et al. Genomic Testing in Patients with Kidney Failure of an Unknown Cause: A National Australian Study. *Clin J Am Soc Nephrol* 2024;**19**:887-97.

8. Blasco M, Quiroga B, García-Aznar JM et al. Genetic Characterization of Kidney Failure of Unknown Etiology in Spain: Findings From the GENSEN Study. *Am J Kidney Dis* 2024;**84**:719-30.e1.

9. Robert T, Raymond L, Dancer M et al. Beyond the kidney biopsy: genomic approach to undetermined kidney diseases. *Clin Kidney J* 2024;**17**:sfad099.

10. de Haan A, Eijgelsheim M, Vogt L et al. Genetic testing in a national cohort of adults with chronic kidney disease of unknown origin. *Nephrology Dialysis Transplantation* 2024;

11. Leenen E, Erger F, Altmüller J et al. Alport syndrome and autosomal dominant tubulointerstitial kidney disease frequently underlie end-stage renal disease of unknown origin—a single-center analysis. *Nephrology Dialysis Transplantation* 2022;**37**:1895-905.

12. Becherucci F, Landini S, Palazzo V et al. A Clinical Workflow for Cost-Saving High-Rate Diagnosis of Genetic Kidney Diseases. *J Am Soc Nephrol* 2023;**34**:706-20.
